# Melatonin Targets Mitochondrial Redox Homeostasis: Optimizing the Intracellular Microenvironment

**DOI:** 10.3390/ijms27104496

**Published:** 2026-05-18

**Authors:** Russel J. Reiter, Ramaswamy Sharma, Doris Loh, Luiz Gustavo de Almeida Chuffa, Yidong Bai, Debora Aparecida Pires de Campos Zuccari, Annia Galano, Walter Manucha

**Affiliations:** 1 Department of Cell Systems and Anatomy, UT Health San Antonio Long School of Medicine, San Antonio, TX 78229, USA; reiter@uthscsa.edu (R.J.R.); baiy@uthscsa.edu (Y.B.); 2 Department of Biomedical Sciences, Baptist University College of Osteopathic Medicine, Memphis, TN 38104, USA; 3 Independent Researcher, Marble Falls, TX 78654, USA; lohdoris23@gmail.com; 4 Department of Structural and Functional Biology, Institute of Biosciences, São Paulo State University (UNESP), Botucatu 18618-689, SP, Brazil; luiz-gustavo.chuffa@unesp.br; 5 Cancer Molecular Research Laboratory (CMRL), Faculdade de Medicina de São José do Rio Preto–FAMERP, São José do Rio Preto 15090-000, SP, Brazil; debora.zuccari@famerp.br; 6 Departamento de Química, Universidad Autónoma Metropolitana-Iztapalapa, Av. Ferrocarril San Rafael Atlixco 186, Col. Leyes de Reforma 1 A Sección, Mexico City 09310, Mexico; agal@xanum.uam.mx; 7 Instituto de Medicina y Biología Experimental de Cuyo (IMBECU), Consejo Nacional de Investigaciones Científicas y Tecnológicas (CONICET), Mendoza 5500, Argentina

**Keywords:** reactive oxygen species, oxidative stress, electron transport chain, biomolecular condensates, lipid rafts, antioxidant, melatonin inducibility, melatonin metabolites, damage radius, liquid–liquid phase separation

## Abstract

The discovery of melatonin as a multifunctional free radical scavenger and its possible synthesis in the mitochondrial matrix of peripheral eukaryotic somatic cells highlights a critical new perspective on the importance of this indole. Experimental evidence supporting these findings is substantial, but there are still lingering questions whether melatonin is a direct radical scavenger in vivo and whether it is synthesized in the mitochondrial matrix. We systematically analyze the innovative experimental approaches that support melatonin’s radical scavenging actions and assess the compelling data supporting its production in mitochondria. Melatonin concentrations are reportedly higher in this organelle than in other cellular compartments. Proteins for the enzymes required to convert serotonin to melatonin are present in the mitochondrial matrix and purified mitochondria synthesize melatonin. In the mitochondrial matrix, melatonin is likely located within the “damage radius” of highly reactive oxygen species. We also summarize novel actions of melatonin associated with its regulation of membrane fluidity, determine the molecular composition of membrane lipid rafts, and modulate liquid–liquid phase separation and biomolecular condensates intracellularly. If the findings discussed herein continue to be validated, melatonin would be in an optimal position to function as an antioxidant and may be a key driver in the context of preserving mitochondrial redox homeostasis and disease mitigation.

## 1. Introduction

The in vivo concentrations of oxygen- and nitrogen-based free radicals, which possess an unpaired electron in their valence orbital, along with non-radical but oxidizing agents, are referred to as either reactive oxygen (ROS) and reactive nitrogen (RNS) species or collectively as free radicals. In the current review, the term free radicals is used interchangeably with ROS or RNS, with the molecular impairments they cause being identified as nitro-oxidative stress or simply oxidative stress (also known as bad stress). It is generally implied that the actions of ROS/RNS are always damaging, which is not the case. Seis [1] introduced the term eustress (or good stress) which occurs when these agents are at low levels and contribute to a variety of biochemical reactions, including hydroxylation and carboxylation, among others, and those involved in signal transduction pathways such as nuclear factor-κB (NF-κB) and nuclear factor erythroid 2-related factor 2 (Nrf2) activation. The current review is particularly directed at bad stress (oxidative stress) associated with endogenously produced free radicals within mitochondria as a result of electron leakage from the electron transport chain, such as from complex I (NADH:ubiquinone oxidoreductase), complex III (cytochrome bc1 complex), and coenzyme Q (ubiquinone) [2,3].

While reactive oxygen- and nitrogen-based reactants are continually produced in low amounts in mitochondria even under basal conditions, they are held in check by a series of direct radical scavengers and antioxidative enzymes, maintaining redox homeostasis and avoiding excessive molecular mutilation. During highly demanding and stressful conditions, however, free radical generation may exceed the antioxidant capacity of the cell such that the unscavenged toxic species mutilate essential molecules, compromising organellar function. Although mitochondria are major sites of free radical generation, they are also produced in other subcellular compartments by a variety of enzymes including oxidases, dehydrogenases, oxidoreductases and others [4,5]. For the current review, however, the emphasis is on free radical activity and oxidative stress that is triggered in the mitochondrial compartment and the means by which endogenously produced and exogenously administered melatonin control their destructive actions.

In addition to providing essential information related to the methodologies used and the data obtained relative to the essential action of melatonin as a radical scavenger at the mitochondrial level, the review was also written to counter the idea that melatonin is not a receptor-independent free radical scavenger. In these contrasting publications, the authors claim that the ability of melatonin to limit oxidative stress is merely a result of the promotion of antioxidative enzymes via actions that also involve its engagement with membrane receptors (so-called MT1 and MT2) or with quinone reductase 2 in the cytosol, an alleged melatonin-binding site [6,7,8,9,10]. Interestingly, a frequently used molecule to chronicle the function of melatonin at the membrane receptor level is the receptor antagonist luzindole, a molecule which itself was shown to be superior to vitamin C and nearly equivalent to melatonin in scavenging the ABTS·^+^, thus challenging the validity of its use to confirm the functional actions of melatonin via formal receptors [11].

Against this background, herein, the current authors summarize the findings from peer-reviewed publications which have applied multiple techniques to document and reaffirm, using pure chemical systems, as well as in vitro and in vivo studies, that melatonin directly scavenges reactive oxygen species and markedly curtails the degree of oxidative stress without reliance on melatonin receptors. In addition to the direct radical scavenging actions that do not depend on the presence of a specific melatonin receptors, recent evidence shows that other critical functions of melatonin, including its ability to modulate the molecular composition and functionality of membrane lipid rafts and the stabilization of cytosolic biomolecular condensates which involve lipid–lipid phase separation, are also independent of the described melatonin receptors [12,13,14].

## 2. Mitochondrion: A Free Radical Factory

Oxygen (O_2_) is required to sustain the survival of aerobic organisms, which comprise an estimated 25% of all animal life forms on Earth, since it is the basis of oxygen-dependent respiration which leads to ATP synthesis. O_2_, however, also gives rise to ROS that mediate molecular damage and contribute to disease processes, especially when they are produced in excess [15,16,17]. Diatomic or ground-state O_2_ is itself a biradical, since it possesses two unpaired electrons each situated in a different π* antibonding orbital; due to this configuration, O_2_ is not highly reactive. The two unpaired electrons are described as having parallel spins, which also allows O_2_ to be paramagnetic.

Inhaled air consists of 21% O_2_, but at rest only about 5% (21% of the total inhaled) is used, with the remainder being exhaled. Its use rises dramatically during intensive exercise (up to 75%). In cells, greater than 95% participates in mitochondrial respiration, where O_2_ serves as the terminal electron acceptor for the electron transport chain (ETC) located in the inner mitochondrial membrane (IMM). The remaining minor portion of O_2_ (1–5%) is utilized by oxidases and other oxygen-requiring enzymes which are primarily located outside the mitochondria. Although O_2_ serves as the parent molecule for ROS, increased use of O_2_ by mitochondria is not necessarily proportional to ROS formation. Free radical generation is impacted by electron leakage from the ETC and not by O_2_ flow, so even with high O_2_ use, ROS production may be minimal. Thus, sometimes the resting state may be associated with greater ROS generation than during exercise. Conversely, conditions such as hypoxia, drug inhibition of the ETC, and high mitochondrial membrane potential lead to elevated free radical formation due to enhanced leakage of electrons from the complexes of the respiratory chain. This leads to redox dyshomeostasis and oxidative stress [18].

Electron leak from the mitochondrial ETC occurs especially but not exclusively at complex I and complex III (free radical “hotspots”), where redox intermediates transiently accumulate and may transfer electrons to O_2_ instead of the intended downstream carriers; this generates the reactant superoxide anion radical (O_2_^∙^^−^) as a byproduct [19,20,21]. At complex I, electrons derived from NADH pass both through the flavin mononucleotide (FMN) site and a series of iron–sulfur clusters before reaching the ubiquinone (Q) binding pocket. However, electron leak can also occur under situations of a high NADH/NAD^+^ ratio, elevations of the membrane potential, or reversal of electron transport from a highly reduced Q pool. When the FMN site and the semiquinone intermediate at the Q-binding channel become excessively depressed, there is an exaggerated probability that O_2_ will acquire an electron and form O_2_^∙^^−^ [22,23]. Complex I usually discharges O_2_^∙−^ into the mitochondrial matrix, where it interacts with antioxidant systems or propagates oxidative stress. High proton motive force can also drive electrons backward, referred to as reverse electron transport, through complex I, generating O_2_^∙^^−^.

Complex III leaks electrons at the Qo site during the Q cycle, where ubiquinol (QH_2_) is oxidized and a semiquinone radical intermediate is formed [20,24,25]. When the semiquinone persists longer than usual—for example, when the Qi site is inhibited, the Q pool is significantly reduced, or electron flow downstream is slowed—O_2_ can capture an electron from this unstable intermediate. In contrast to complex I, complex III releases O_2_^∙−^ into both the matrix and into the intermembrane space (IMS) and eventually influences cytosolic signaling pathways. While lower-flux leak sites exist in dehydrogenases which feed electrons into the Q pool, complexes I and III are dominant physiologically in reference to ROS production [26].

Overall, electron leak arises when redox centers become over-reduced, electron flow becomes bottlenecked, or semiquinone intermediates persist long enough for O_2_ to act as an unintended electron acceptor, making ROS generation an intrinsic, regulated consequence of mitochondrial respiration.

Major mitochondrial toxins include rotenone, antimycin A, cyanide, paraquat, and doxorubicin, etc., all of which disrupt electron transport and dramatically augment ROS formation [27,28]. Rotenone blocks complex I, causing electron backflow and O_2_^∙−^ generation. Antimycin A is a complex III inhibitor; it stabilizes the semiquinone that leaks electrons to O_2_. Cyanide impedes complex IV, forcing upstream complexes and causing a highly reduced, ROS-producing state. Paraquat undergoes redox cycling at complex I, continuously generating O_2_^∙−^. Doxorubicin binds cardiolipin, a major phospholipid in the IMM, and redox cycles with mitochondrial enzymes, amplifying O_2_^∙−^, as well as its downstream radical products. Together, these toxins destabilize redox balance and drive oxidative mitochondrial injury.

**Figure 1:**
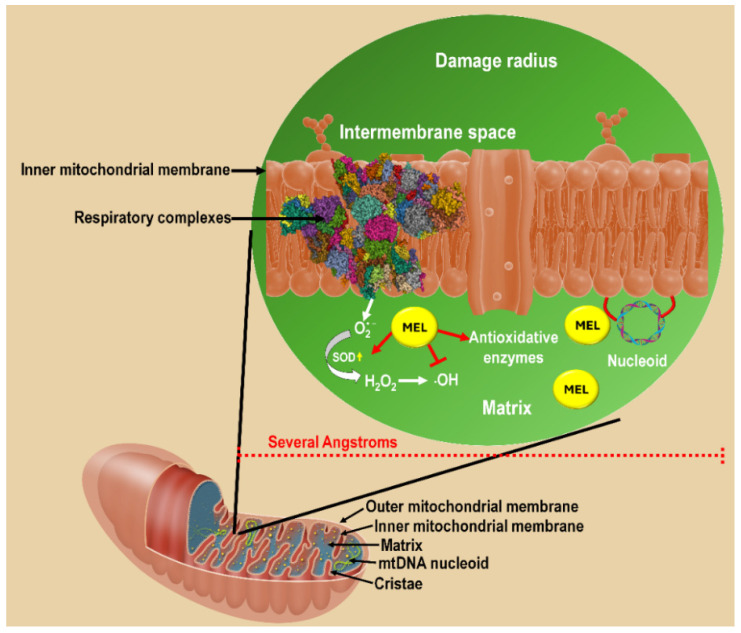
**The area (damage radius) within which a free radical must be scavenged to prevent damage.** Due to the ultrashort half-life of free radicals and the short distance they move (several angstroms) before damaging a molecule or being scavenged, the area in which these events occur is sometimes referred to as the “damage radius” (or “reaction sphere”), as illustrated in this figure. Because of these physiological features, for a scavenger to incapacitate a radical it must also be in the “damage radius”. Since the antioxidant melatonin (MEL) is synthesized in the mitochondrial matrix it, and its antioxidant derivatives, would also be in this space. If not scavenged, the locally produced radicals damage phospholipids in the inner mitochondrial membrane (IMM), proteins and the mtDNA-packaging protein complexes (nucleoid) which are tethered to the IMM. Also, melatonin upregulates antioxidative enzymes which aids in reducing oxidative stress.

Free radicals have extremely short half-lives, often lasting only picoseconds to nanoseconds before reacting with nearby molecules or being neutralized [29]. For example, the highly reactive hydroxyl radical (·OH) and peroxynitrite (ONOO^−^) essentially only damage molecules at the site of their formation (within a couple of angstroms) [30]. Their fleeting existence reflects their high reactivity, as unpaired electrons drive them to seek rapid stabilization through oxidation, reduction, or radical–radical recombination. In biological systems, this short half-life localizes their effects, meaning damage occurs close to the site of formation (sometimes referred to as the “damage radius”), shaping both their signaling roles and their contribution to oxidative stress. In mitochondria, the damage radius is impacted by the degree of protein crowding, redox-active metal locations, membrane proximity and antioxidant buffering capacity. As an example, the highly reactive ·OH essentially only damages molecules at the location of its formation and is identified as onsite damage [31,32]. Thus, any scavenger that can neutralize these reactants must be situated in the “damage radius” (Figure 1). Recent integrative reviews emphasize that mitochondria-derived ROS drive disease pathophysiology through persistent redox imbalance, positioning mitochondria as central regulators of oxidative stress in metabolic, cardiovascular, and neurodegenerative disorders [33].

## 3. Melatonin: Silence of the Radicals

Consistent with the presumed exclusive production of pineal melatonin during the night and its dormant synthetic activity during the day, for the initial 35 years after its discovery, melatonin was identified as an essential circadian [34] and circannual [35] regulator functioning as both a clock and as a calendar [36]. Melatonin’s known functional repertoire changed abruptly when, in 1993, what proved to be an evolutionary conserved function of the indoleamine was uncovered. This previously unsuspected action of melatonin that was discovered was its function as a free radical scavenger [37,38]. At this time, the pineal gland was thought to be the primary source of melatonin in vertebrates even though it had been found to be synthesized in Drosophila and other insects which lack a pineal homolog [39]. The suspicion that melatonin may be capable of neutralizing free radicals was prompted by an observation that was made related to melatonin’s ability to protect DNA from damage by the chemical carcinogen safrole [40].

### 3.1. Electron Spin Resonance Spectroscopy Studies

**Figure 2:**
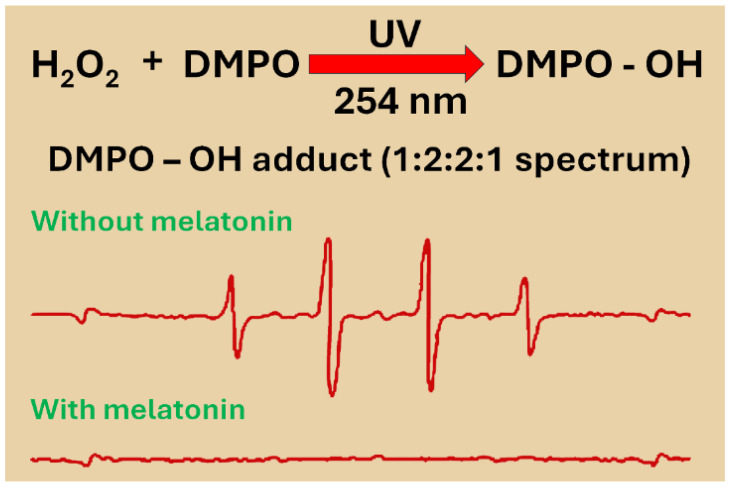
**Electron resonance spectroscopic (ESR) analysis of melatonin’s ability to quench the hydroxyl radical in a purely chemical setting.** A solution of hydrogen peroxide H_2_O_2_ and the spin trapping agent 5,5-dimethyl-1-pyrroline-N-oxide (DMPO) was exposed to ultraviolet light (UV) at 254 nm, causing the peroxide to photolyze to generate hydroxyl radicals which were trapped by DMPO, producing the adduct DMPO-OH. DMPO-OH adducts have a long half-life and exhibit a characteristic 1:2:2:1 spectrum on ESR. The addition of melatonin to the system quenched the DMPO-OH ESR signal, indicating that it had scavenged the hydroxyl radicals preventing the formation of DMPO-OH adducts. Figure adapted from [46].

Verification of this novel free radical scavenging action of melatonin required compelling proof. To supply that evidence, electron spin resonance spectroscopy (ESR), the most widely accepted method for documenting the scavenging activity of any molecule, was employed [41]. ESR functions by recognizing transitions between the levels of magnetic energy of unpaired electrons (paramagnetic species) when they are exposed to microwave irradiation in a magnetic field [42]. To achieve this, a solution of hydrogen peroxide (H_2_O_2_) was photolyzed by exposure to ultraviolet (UV) light, which resulted in the formation of the ·OH. The extremely high reactivity of the ·OH causes this species to have an ephemeral half-life (in the nanosecond range), making it difficult to directly measure [43]. To stabilize the ·OH, a spin trapping agent which yields a spin adduct that decays over a longer period of time (in hours) was used, making the radicals indirectly measurable with ESR. To assess the ·OH, we utilized the spin trapping agent 5,5-dimethyl-1-pyrroline-N-oxide (DMPO), which is commonly used for such estimations [44]. The DMPO-·OH adduct was detected by high-performance liquid chromatography with an electrochemical detector (HPLC-EC) which was validated by ESR. The ESR signal for the DMPO-·OH adduct yielded the characteristic and specific 1:2:2:1 spectrum [45]. When melatonin was supplemented to the DMPO-·OH-generating system, the ESR signal was quenched in a dose-dependent manner (Figure 2). For verification, in separate studies, rather than melatonin, either glutathione or mannitol (both established free radical scavengers) were added to the DMPO-·OH mixture. Their ability to alter the ESR signal proved to be weaker than that of melatonin. In this system, the concentrations of each molecule to inhibit the ESR signal by 50% (IC_50_) were 21, 123 and 273 μM for melatonin, glutathione and mannitol, respectively, indicating that melatonin is superior to the other antioxidants relative to scavenging the ·OH [38]. Also, since melatonin quenched the ESR signal in a cell-free system, this action of melatonin was obviously receptor independent.

The publication of this new and unanticipated function of melatonin was initially viewed with considerable skepticism. Thus, confirmation of these findings was critical, and this was soon accomplished by Bromme and colleagues [47] who also used ESR spectroscopy to certify ·OH scavenging by melatonin. In an in vitro system, this group used the spin trap 5-(diethoxyphosphoryl)-5-methyl-1-pyrroline-N-oxide (DEPMPO) in a solution where the ·OH was generated in a reaction mixture of alloxan with glutathione and ferrous iron. Melatonin quenched the ESR spectrum that identified the ·OH with an IC_50_ value of 23 μM, a value like that we had reported [38]. In the same publication, Bromme and colleagues [47] found that melatonin also reduced the accumulation of malondialdehyde (MDA), an oxidative byproduct of polyunsaturated fatty acid (PUFA) degradation, in phosphatidylcholine-containing liposomes in the same radical-generating system.

Molecules with structural similarities to melatonin were also soon investigated for their efficacy in scavenging the ·OH [48]. In addition to melatonin, the molecules tested included 5-hydroxytryptamine (5-HT, serotonin), 5-methoxytryptamine (5-MT), 6-chloromelatonin (6-cMEL), and kynuramine (KN). In this report, the ·OH was generated using Fenton reagents (H_2_O_2_ and ferrous iron) and the spin trap DMPO, with the results being evaluated with ESR. Matuszak and colleagues [48] calculated the rate constant for melatonin with ·OH as 2.7 × 10^10^ M^−1^s^−1^ at pH 7.0. The other indoles exhibited equally high-rate constantsin the range of 10^10^ M^−1^s^−1^. Because of the data available at the time, i.e., blood levels of melatonin typically being in the low nM range even at night, the authors surmised that in vivo melatonin would be a minor free radical scavenger. They based their estimates, however, on blood concentrations of melatonin, which are irrelevant in terms of the intracellular scavenging activity of a molecule. Moreover, more recent studies have documented that melatonin is produced in the mitochondria of probably every cell, a site where free radical generation is often at its maximum [49]. Thus, intracellular melatonin is present at significantly higher measured concentrations than levels in the blood [50] and is critically situated to neutralize locally metabolically generated mitochondrial radicals [51].

### 3.2. Pulse Radiolysis Studies

Stasica and co-workers [52] used a different technique to estimate the efficacy of melatonin as a ·OH scavenger. Pulse radiolysis is an effective method for measuring antioxidant scavenging [47]. Pulse radiolysis generates short-lived radicals which can be neutralized by scavengers in a nanosecond-to-microsecond time range. The information garnered provides data on both the mechanisms and the rate constants of the scavenging actions. Using this method, melatonin’s interaction with the ·OH was estimated to have a rate constant of 1.2 × 10^10^ M^−1^s^−1^ [53]. Based on these findings, they concluded that melatonin is a highly potent ·OH scavenger.

**Figure 3:**
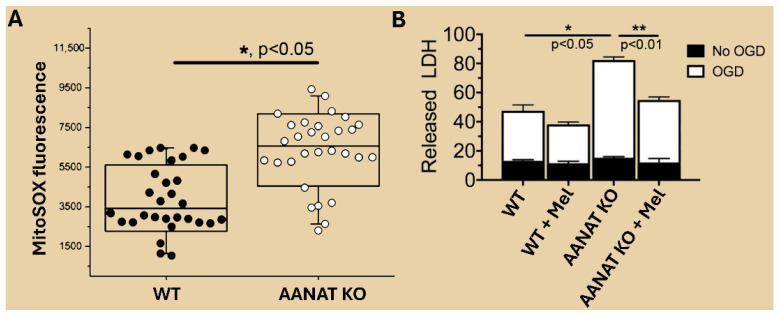
**Evidence related to melatonin’s scavenging of O_2_^∙−^ in isolated neural mitochondria.** The rate-limiting enzyme arylalkylamine N-acetyltransferase was knocked out (AANAT KO) in mitochondria of mouse neuroblastoma Neuro-2a (N2a) neurons. MitoSOX^®^, a specific indicator of O_2_^∙−^, is capable of entering mitochondria since it is coupled with triphenylphosphonium cation (TTP^+^) which drives it into the mitochondria. When intramitochondrial melatonin was diminished due to ablation of AANAT, O_2_^∙−^ accumulated in larger amounts, as evidenced by increased fluorescence compared to the levels in mitochondria in which melatonin synthesis was not reduced even in the absence of a challenge with a free radical stimulating agent (**A**). The figure on the right (**B**) illustrates the release of lactate dehydrogenase (LDH) from wild-type (WT) or knocked out AANAT N2a neurons that were subjected to oxygen/glucose deprivation (OGD), in an in vitro model of stroke, or not. OGD stimulates free radical generation which damages cells and enhances LDH release. As seen, this was inhibited by melatonin. These cells also exhibited elevated cytochrome c release and caspase 3 activity, both of which were also suppressed by supplementation with melatonin. Figure was adapted with permission [56].

Roberts and colleagues [54] also used pulse radiolysis to evaluate the scavenging efficiency of melatonin against both ·OH and O_2_^∙−^. They observed that melatonin neutralized the ·OH with a rate constant of 1.3 × 10^10^ M^−1^s^−1^, which approximates the diffusion-controlled rate. Conversely, melatonin in this system proved ineffective as an O_2_^∙−^ scavenger. The authors also pointed out that the ·OH only travels a maximal distance of three angstroms before interacting with another molecule [30,55]. On the basis of what was known at the time of their report regarding the intracellular concentration and location of melatonin within cells, the authors seemed reluctant to characterize melatonin as a noteworthy antioxidant in vivo. However, as already noted above, melatonin levels are present at much higher concentrations in some cellular organelles than in the blood, and there are especially high levels in the mitochondria, the site of elevated free radical generation [50]. Moreover, later studies by Suofu and colleagues [56] have confirmed that melatonin is highly effective in reducing free radical generation in isolated mitochondria despite the short distance they move before damaging a neighboring molecule (Figure 3). Thus, melatonin must be in the immediate vicinity of where the oxidizing radicals were formed to reduce their destructive actions.

### 3.3. In Vitro Studies

Soon after melatonin was discovered as a OH scavenger, Marshall and colleagues [57] performed a series of in vitro investigations to identify its actions against other reactive species. Melatonin proved effective in protecting catalase and preventing the oxidation of 5-thio-2-nitrobenzoic acid from the toxicity of hypochlorous acid (HOCl), a potent oxidizing agent. Although HOCl is not produced in mitochondria, these organelles are highly vulnerable to this agent when it enters cells and induces cardiolipin oxidation in the IMM, leading to mitochondrial permeability transition pore (mPTP) opening. Also, melatonin limited the peroxidation of brain-derived phospholipids and reacted with the trichloromethylperoxyl radical (CCl_3_O_2_^·^) but failed to effectively scavenge the O_2_^∙−^ and exhibited limited efficacy in protecting DNA from damage during exposure to a ferric bleomycin system.

The reported limited efficacy of melatonin to neutralize the O_2_^∙−^ is consistent with similar observations made by Roberts and colleagues [54] using a different assay system. Without knowledge of its intracellular concentrations or its subcellular distribution in intact cells, actions which were uncovered years later [50,56], Marshall and co-workers [57] surmised that endogenously produced melatonin, because of its low levels, may provide only modest antioxidant protection in vivo, a view that currently runs contrary to numerous reports documenting its robust ability to reduced oxidative stress, including oxidative damage to DNA, under countless in vivo circumstances [58,59].

A combination of proton nuclear resonance, carbon nuclear magnetic resonance and mass spectrometry was used to verify that melatonin is directly involved in the removal of H_2_O_2_ [60]. H_2_O_2_ is toxic when it is in high concentrations in cells, is the precursor of the ·OH, and has a relatively long half-life (in seconds), allowing it to diffuse between cells and thereby initiate molecular damage far from its site of production [61]. It is normally metabolized by catalase and glutathione peroxidase to water. Additionally, however, it is detoxified by melatonin, helping to keep intracellular H_2_O_2_ concentrations at a steady-state level.

### 3.4. Biochemical Studies in Cell-Free Systems

**Figure 4:**
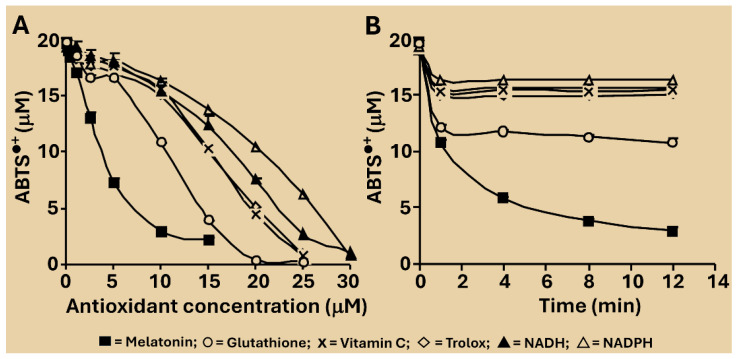
**Comparative study related to the efficiency of melatonin and other classic antioxidants in scavenging ABTS·^+^.** The antioxidants include melatonin, reduced glutathione, vitamin C, Trolox, NADH, and NADPH. (**A**) Dose dependence of individual scavengers against concentrations of ABTS^·+^ ranging from zero to 20 μM measured after a 12 min incubation period. In this pure chemical system, melatonin was clearly superior to the other five agents in neutralizing the cation radical. (**B**) Time dependence curves for each antioxidant at a concentration of 10 μM at several time intervals after initiation of incubation. The pattern of scavenging by melatonin obviously varies from that of the other scavengers. Each of the scavengers, except melatonin, were depleted within 1 min (the earliest time point recorded) following exposure to ABTS·^+^. Melatonin continued to scavenge radicals for 12 min. This continuous scavenging pattern was likely a consequence of intermediates that were formed and are likewise radical scavengers. The legend identifying each of the scavengers is listed under the figure. For additional details, the reader can consult the text. Data restructured from [62].

The ability of melatonin to scavenge radicals in a purely chemical setting in comparison to classic antioxidants also was addressed in a report we published in 2003 [62]. For this purpose, we used the ABTS cation radical (ABTS·^+^), a stable, intensely blue-green reactant that can be readily monitored spectrophotometrically at an absorbance of 734 nm. Antioxidants donate either electrons or hydrogen atoms to ABTS·^+^, thereby reducing it to ABTS. The radical scavengers that were compared to melatonin included reduced glutathione (GSH), Trolox (a water-soluble vitamin E analog), vitamin C, NADH and NADPH. The results revealed that melatonin scavenged the cation radical with an IC_50_ of five, while the other agents had IC_50_ values of 11, 15.5, 15.5, 17 and 21 for GSH, Trolox, vitamin C, NADH and NADPH, respectively. In this system, the classic antioxidants scavenged one or fewer ABTS·^+^, while melatonin detoxified an estimated two to four radicals (Figure 4). A supplemental cyclic voltammetry measurement indicated that melatonin transfers an electron at a voltage of 715 mV. Melatonin’s scavenging actions indicated that it involved multiple-electron donations with the possible generation of intermediates, presumably including cyclic 3-hydroxymelatonin (cyclic 3-OHM) and N_1_-acetyl-N_2_-formyl-5-methoxykynuramine (AFMK) [62]. Due to the formation of these intermediates, a cascade of reactions allows melatonin (via its metabolites) to scavenge more than a single ABTS·^+^.

**Figure 5:**
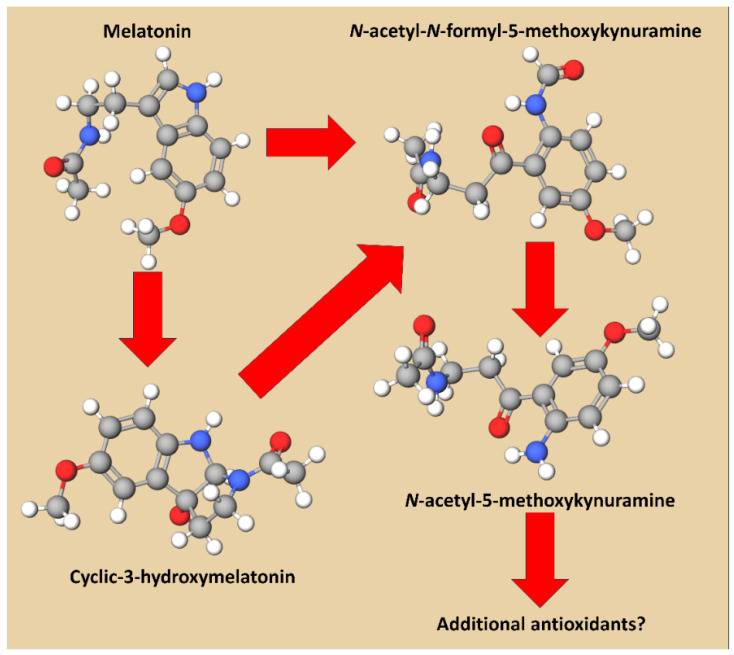
**A summary of melatonin’s radical scavenging cascade.** This is a non-enzymatically driven process resulting from ROS reduction. When melatonin detoxifies a reactive species, it is converted to the intermediate molecule cyclic 3-hydroxymelatonin, which is itself a scavenger resulting in the formation of AFMK. Subsequently, AFMK is likewise a scavenger, and in the process, it is further oxidized to AMK with additional downstream molecules perhaps being generated. Some of the metabolites of melatonin, and depending on the radical that is neutralized, are comparable to or more effective scavengers than melatonin itself [66,67]. Melatonin is also directly metabolized to AFMK by the enzyme indoleamine 2,3-dioxygenase.

The curve for the scavenging of ABTS·^+^ also differs from that of the other antioxidants with which it was compared. As shown in Figure 4B, melatonin continued to reduce the level of ABTS·^+^ for at least 12 min after these agents were mixed. Moreover, 10 μM of melatonin neutralized a 20 μM concentration of the cation radical. These observations suggested that the metabolite(s) of melatonin generated as a consequence of its interaction with ABTS·^+^ may also quench this agent. One of those metabolites, which proved to be an early intermediate in the so-called antioxidant cascade (Figure 5), was identified as cyclic 3-hydroxymelatonin (3-OHM), which is essentially one “footprint” of radical scavenging by melatonin [63]. 3-OHM reacts with ·OH at a diffusion-limited rate [64]. Additionally, 3-OHM is a highly efficient ROO· scavenger, making it a better chain-breaking antioxidant than melatonin, a point at which melatonin has weaker scavenging activity [65].

### 3.5. Oxygen Radical Absorbance Capacity Studies

Sofic and co-workers [68] employed the oxygen radical absorbance capacity (ORAC) assay to assess the efficacy of melatonin and other antioxidants as radical detoxifying agents. For the measurements, 2,2-azobis(2-amidino-propane) dihydrochloride (AAPH) was used to generate the peroxyl radical (ROO·), while a combination of H_2_O_2_ and Cu^2+^ generated the ·OH. In these systems, melatonin was compared with GSH, vitamin C and Trolox relative to their abilities to quench the ROO· and ·OH. Melatonin was highly effective in neutralizing both the ROO· and ·OH and, moreover, it was significantly better than GSH, vitamin C or Trolox in doing so [68]. Scavenging of the ROO· has relevance to mitochondria since the composition of both the OMM and the IMM are rich in readily oxidizable phospholipids, including the critically important cardiolipin. Because of their findings, the authors claimed that melatonin may be a premier molecule to protect against free radical damage [68].

### 3.6. Studies of Melatonin as a Scavenger of Other Toxic Reactants

O_2_ is converted to an excited high-energy form by the addition of energy, with the product being referred to as singlet oxygen (^1^O_2_). This oxygen derivative is biologically toxic, with the ability to rapidly oxidize essential biomolecules. For example, it reacts with polyunsaturated fats, leading to lipid peroxidation, and enzymes containing reactive cysteine residues are also readily damaged by ^1^O_2_ [69]. While the association between melatonin and ^1^O_2_ has been sparingly investigated, there is one report that provides indirect evidence for a role of melatonin in neutralizing this high-energy oxygen state [70].

Nitric oxide (NO^∙^), a reactive nitrogen species (RNS), is a free radical gaseous neurotransmitter which at high concentrations has some toxicity, especially to the respiratory system when inhaled and when it is in elevated levels in cells [71]. It also couples with O_2_^∙−^ to form the highly reactive and potent oxidizing agent peroxynitrite (ONOO^−^) [72]. The Griess reaction is a classic analytical chemical method for detecting and quantifying nitrite (NO_2_^−^). Noda and colleagues [73] compared the efficacy of melatonin with 5-hydroxytryptophan and L-tryptophan to neutralize NO^∙^ in the Griess reaction, as evidenced by the reduction in NO_2_^−^. The study revealed that, of the three molecules considered, melatonin was the most potent scavenger of NO^∙^. From the standpoint of oxidative stress, while reducing the NO^∙^ level is important [74], its depletion, which results in reduced generation of the ONOO^−^, is also highly relevant since the latter is considered to be on a par with the ·OH in terms of inducing molecular damage [75]. ONOO^−^ is a potent oxidizing and nitrating agent which can contribute to mitochondrial dysfunction. While melatonin has been proposed as a direct ONOO^−^ and/or a peroxynitrous acid (ONOOH) scavenger, the evidence supporting this claim is not as complete as for the oxygen-based reactive species [76].

While the tests performed and summarized herein are generally supportive of melatonin being a multifunctional antioxidant, its efficiency in scavenging the O_2_^∙−^ varied somewhat according to the methodology used for evaluation. While the preponderance of evidence indicates that melatonin does neutralize O_2_^∙−^, subsequent studies should further define this action. For example, an early metabolite in melatonin’s antioxidant cascade, cyclic 3-hydroxymelatonin, is readily capable of neutralizing O_2_^∙−^. An important consideration is the reliability of the methods used for the measurements.

Although published reports leave little doubt that melatonin directly neutralizes a number of radical species in the conditions used in those studies, proving that these reactions occur in vivo is far more difficult. This is an issue with all radical scavengers. Generally, their efficacy in vivo is determined by biochemical footprints that are formed, such as for vitamin C, the oxidized product dehydroascorbic acid. For melatonin, the biochemical footprints are cyclic 3-hydroxy melatonin, AMK and AFMK. This evidence provides chemical plausibility but does not definitively establish physiological relevance.

## 4. Melatonin’s Offspring as Radical Scavengers: Chemical Evidence

Downstream molecules that are formed when melatonin functions as a scavenger are also effective in this capacity and are sometimes more efficient in neutralizing ROS than is the parent molecule; we named this sequential scavenging melatonin’s antioxidant cascade [77]. Cyclic 3-hydroxymelatonin rapidly neutralizes ·OH and ROO·, exhibiting diffusion-limited kinetics and potent antioxidant activity within the melatonin cascade, enhancing cellular protection against oxidative stress. Also, AFMK and N1-acetyl-5-methoxykynuramine (AMK), two major kynuramine-pathway metabolites of melatonin, exhibit exceptional efficacy as radical scavengers across diverse biological environments. AFMK efficiently neutralizes ·OH, ROO·, and ^1^O_2_ species, acting as a versatile antioxidant that interrupts radical-driven chain reactions. AMK is even more potent: its electron-rich indole–quinone structure enables rapid single-electron transfer and hydrogen-donation reactions, allowing it to quench highly reactive species such as ·OH, ·NO, ONOO^−^, and lipid-derived ROO· [78]. Both metabolites display reaction rates that surpass melatonin itself, extending the antioxidant cascade initiated by the parent molecule. Their amphiphilic properties allow activity in aqueous and lipid compartments, enhancing the protection of membranes, proteins, and nucleic acids. Together, AFMK and AMK function as durable, multitarget radical scavengers that likely amplify melatonin’s overall antioxidant capacity and sustain protection during prolonged oxidative stress [79,80,81]. The cascade effectively increases the concentration of melatonin within cells.

Electron leakage and generation of O_2_^∙−^ at complexes I and III are impacted by the mitochondria membrane potential. By stabilizing the membrane potential, melatonin regulates uncoupling protein 2 (UCP2) and limits electron leakage such that oxidative stress is held in check, which aids in improved cellular survival [82,83,84].

## 5. Quantum-Mechanical Calculations Confirm Melatonin as a Radical Scavenger

To provide additional insights into the mechanism by which melatonin interacts with radicals, several groups carried out computational studies. Stascia and colleagues [85] examined potential intermediates and end products after melatonin interacts with the ·OH. Their theoretical calculations suggested that carbon atoms at two, three, six and seven of the indole ring would likely interact with the ·OH, with kinetic evaluations suggesting C2 may be the most likely site for the ·OH to target. Likewise, thermodynamically, the melatonin–OH adduct with the ·OH at C2 was more stable than the others. The computer calculations are in line with predictions that melatonin readily reacts with the ·OH, with the resulting formation of a melatonin–OH adduct.

Using semiempirical calculations, the product formed when melatonin interacts with NO^∙^ was identified as N-nitrosomelatonin [86]. Two mechanistic pathways were examined, the first being a radical process that involved hydrogen ion abstraction accompanied by the addition of NO^∙^. The second calculation included the nitrosonium ion interacting with the indole moiety. In essence, N-nitrosomelatonin is a product of a radical process in which NO^∙^ is added to a melatonin radical. The findings suggest that the indole nitrogen is the most likely site of the nitrosation of NO^∙^ involving a radical mechanism.

Additional mechanisms of melatonin and structurally related molecules as radical scavengers have been identified using different computational tools. These mechanisms include: (I) Direct radical scavenging, generally known as primary antioxidant activity (AOX-I or interception). AOX acronyms used in this section relate to systems biology models of oxidative stress and redox signaling network simulations [87,88,89]. AOX-I either yields a non-damaging radical or terminates a chain reaction. (II) Secondary antioxidant behavior (AOX-II) relies on inhibition of ·OH generation by sequestering redox metals, e.g., Cu^2+^ and Fe^3+^, thereby interrupting the Fenton reaction. (III) Tertiary antioxidant activity (AOX-III) involves the repair of oxidatively damaged biomolecules to their pristine form. (IV) Includes regulation of the activities of enzymes that change the redox environment. These are known as enzyme modulators or AOX-E. These actions can include promotion of antioxidative enzymes, inhibition of pro-oxidative enzymes or modulation of transcription factors (TFs).

**Figure 6:**
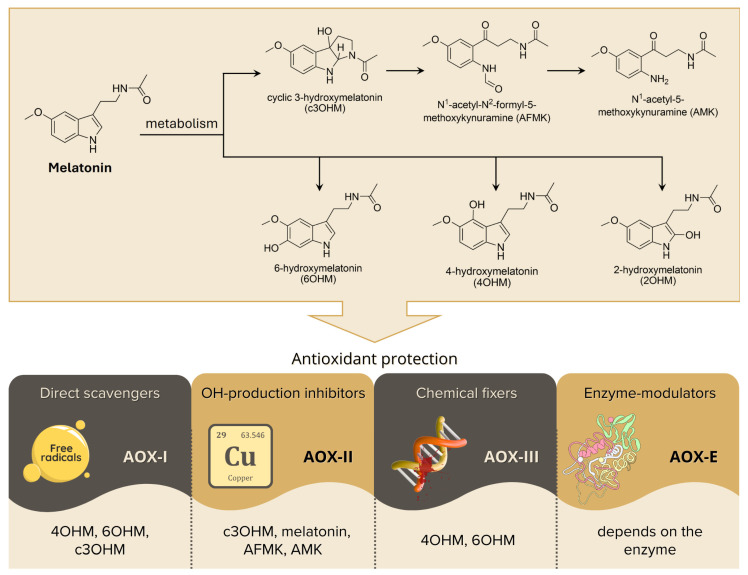
**Mechanisms involved in modulating oxidative stress by melatonin and its metabolites.** Metabolic pathways (**top**) resulting in the formation of the molecules that function in each of the categories (**bottom**) identified, as based on the outcomes of published computational studies, are shown. These data summarize the ability of different indoleamines to reduce radicals in each of the categories noted, as estimated only by computational studies. The figure does not include data from the chemical analyses presented in earlier sections of this review. AOX-I is based on the rate constants for the reactions between the investigated compounds and peroxyl radicals (ROO·). In addition, they were compared with those corresponding to the reaction with Trolox, the reference antioxidant, with the same radical. 4-Hydroxymelatonin (4OHM), 6-hydroxymelatonin (6OHM), and cyclic 3-OHM scavenged ROO· ~206, 40 and 32 times faster than Trolox, respectively, in aqueous solution, at pH = 7.4. This trend is influenced by the environment, especially for c3OHM, which in lipid media becomes less reactive than Trolox towards ROO·. In reference to AOX-2, the most straightforward means to analyze the calculated data in this category is thermochemically based methods. The thermochemical viability of metal reduction depends on the strength of the reductant, two of which were utilized in these calculations, i.e., the O_2_^∙−^ and Asc^−^. Based on the results, cyclic 3-OHM, melatonin and N1-acetyl-N2-formyl-5-mehthoxyknuramine (AFMK) were deemed the most efficient scavengers. The AOX-III molecular repair pathways involve several processes depending on how the specific biomolecule was oxidatively damaged. For these estimates, lesions induced in 2-deoxyguanosine due to (a) loss of a single electron from the guanine moiety yielding the corresponding radical cation, (b) loss of a hydrogen atom in the sugar moiety leading to C-centered radicals, and (c) the formation of a DNA adduct, typically at the C8 site in guanine; this common lesion is identified as 8-hydroxy-2-deoxyguanosine. For (a), melatonin and its metabolites were all predicted to be effective chemical fixers utilizing single-electron transfer (SET), formal hydrogen atom transfer (fHAT), and sequential hydrogen atom transfer dihydration (SHATD). The estimated rate constants (*k*) for each of these reactions were on the order of 10^9^ M^−1^ s^−1^. For (b), 4OHM was identified as the most promising repair agent (*k* = 5.32 × 10^2^ M^−1^ s^−1^). For (c), 4OHM was again found to be the most rapidly repairing agent, with *k* = 5.32 × 10^2^ M^−1^ s^−1^; 6OHM had *k* of 1.06 × 10^4^ M^−1^ s^−1^. AOX-E depends heavily on the enzyme being investigated. Regarding the upregulation of antioxidative enzymes, 4OHM was most effective as a catalase inducer. Based on molecular docking simulations, several derivatives inhibited pro-oxidative enzymes. In particular, cyclic 3OHM and AFMK interact with the proline-rich region peptide (p47^phox^); AFMK and AMK suppress neuronal nitric oxide synthase (nNOS), while melatonin, cyclic 3OHM, 4OHM and 6OHM reduce the activity of thioredoxin reductase (TrxR). In reference to transcription factor (TF) regulation, the calculations revealed that melatonin, AFMK, and AMK were predicted as modulators of the peroxisome proliferator-activated receptor alpha (PPARα); 4OHM, 6OHM, and AMK are modulators of proliferator-activated receptor gamma (PPARγ); and cyclic 3OHM is a modulator of Kelch-like ECH-associated protein 1 (KEAP1).

Mechanisms (I), (II) and (III) were investigated using the QM-ORSA (Quantum Mechanics-based-test for overall free Radical Scavenging Activity), a protocol which is based on Density Functional Theory (DFT) and conventional transition state theory (TST) [90]. This protocol allows for assessing thermochemistry, and estimating overall rate constants, taking into account the effects of solvent polarity, pH, speciation, tunneling effects, and diffusion limits. Mechanism (IV) was explored using molecular docking and molecular dynamics, with the analyses including not only the binding energies but also a similarity interaction score (S_SI_) meant to quantify the resemblance of the pose of a particular ligand in the enzyme pocket with that of a reference compound known to have the desired effect. The results of these computational analyses are consistent with the observed actions of melatonin in reducing redox dyshomeostasis in in vivo studies. The efficacy of melatonin and its metabolites in modulating oxidative stress via these mechanisms varies, as summarized in Figure 6.

The computational calculations and simulations suggest that important complementary actions of melatonin and its metabolites modulate multiple functions that control the degree of oxidative stress that normally accumulates in all subcellular compartments, including in the mitochondria. These calculations are consistent with what is identified as the antioxidant cascade of melatonin. Additional details related to the derivation of the data using computational methods can be found in several publications [58,90].

While the data from ESR spectroscopy, pulse radiolysis and theoretical calculations are compelling in support of melatonin as a radical scavenger, they are not indisputable since these studies measured melatonin’s ability to neutralize the ·OH under in vitro conditions or under simulated circumstances. In the last 25 years, however, it has been unequivocally established that melatonin mitigates free-radical-driven oxidative stress mediated by each of the ROS mentioned in this report under numerous experimental and clinical conditions [65,91,92].

## 6. Melatonin and Redox Imbalance: Relation to Mitochondrial Biomolecular Condensates

Liquid–liquid phase separation (LLPS) is a rapidly emerging subject of interest in free radical biology, as well as in melatonin’s involvement in processes related to formation of membrane lipid rafts and cytosolic biomolecular condensates (BCs) [93,94]. The process of LLPS modulates free radicals and has potential clinical applications [95]. The mitochondrial matrix is a distinct biophysical environment where intrinsic macromolecular crowding conditions facilitate the rapid assembly of functional BCs via phase separation. The level of oxidative stress in closely linked to the aggregation and dissolution of BCs [96]. Changes in cellular environments, including temperature, pH, and ion gradients, also affect the thermodynamic regulation of phase separation. BC formation and dissolution are tightly regulated by multivalent intermolecular interactions and the system’s overall free energy [97]. Under physiological conditions, high matrix viscosity acts as a requisite constraint, reducing the mean free path to promote the weak, multivalent interactions necessary for dynamic, fluid assemblies [98,99]. This material state is critical for mitochondrial quality control, particularly mitophagy, where damaged organelle sequestration requires rapid spatiotemporal organization of autophagy receptors. Mitophagy-associated proteins, p62/SQSTM1, NDP52 (CALCOCO2), and optineurin (OPTN), are redox sensors that phase separate to cluster ubiquitinated mitochondrial cargo and recruit autophagosomal machinery [100]. Condensate fluidity allows for efficient exchange of signaling factors, ensuring a rapid and reversible mitophagic response. However, this dependence renders the quality control system highly susceptible to alterations in the physicochemical properties of the matrix solvent, particularly those induced by ROS.

Redox imbalance potently regulates mitochondrial condensate dynamics, driving the matrix toward pathological kinetic arrest [96]. While physiological viscosity supports ergodic fluid assemblies, excessive ROS alters matrix properties. Mechanistically, the negative polarity of the hydroxyl radical (·OH) allows it to act as a hydrogen bond acceptor, stabilizing water networks and elevating effective viscosity in the matrix’s interfacial water [94]. This viscosity amplification, alongside oxidative modifications to protein multivalency, accelerates BC aging, a rheological transition from ergodic Maxwell fluids to kinetically arrested Kelvin–Voigt solids or glassy states [99,101]. Aged, kinetically arrested BCs are functionally compromised as they fail to dynamically tether to autophagosomal membranes and instead sequester essential signaling factors in non-productive assemblies [102]. Consequently, redox-induced mitophagy failure represents a biophysical phase-transition breakdown driven by increased viscosity within both the BCs and the mitochondrial matrix.

**Figure 7:**
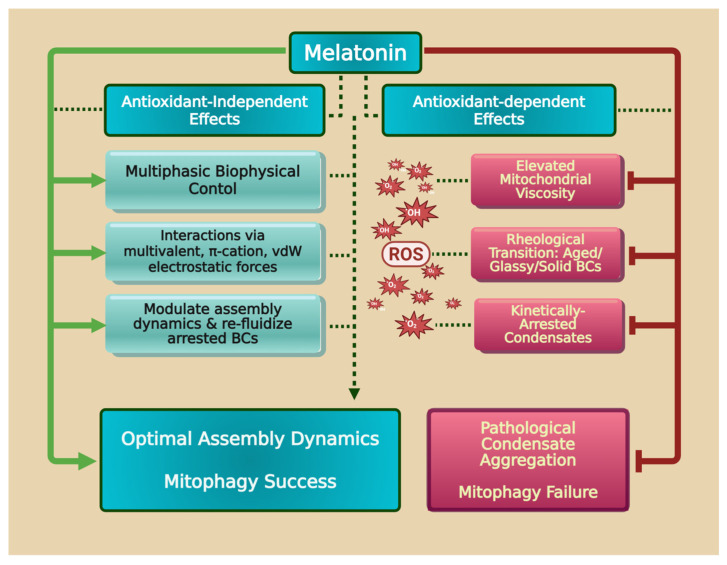
**Multiphasic biophysical control of mitochondrial mitophagy proteostasis by melatonin resulting from its antioxidant actions.** This flowchart delineates the multiphasic regulation of melatonin over mitochondrial biomolecular condensates (BCs). (**Right**) Antioxidant-dependent axis: Melatonin buffers the redox environment by neutralizing reactive oxygen species (ROS), thereby maintaining mitochondrial matrix viscosity within a physiological window permissive for ergodic, fluid phase separation. In the absence of this regulation, ROS-mediated viscosity amplification triggers a rheological transition from fluid Maxwell assemblies to kinetically arrested, glassy, or solid states, resulting in mitophagy failure. (**Left**) Antioxidant-independent axis: Melatonin exerts direct biophysical control over BC scaffolds, utilizing multivalent, electrostatic, and van der Waals forces. Also, melatonin interferes with condensate-interacting regions to modulate assembly kinetics to protect condensate functionality. (Figure created in https://BioRender.com).

Melatonin exerts homeostatic, context-dependent modulation of mitophagy, protecting cellular integrity via pleiotropic molecular mechanisms. It enhances clearance of toxic aggregates [103,104] while suppressing maladaptive mitophagy under pathological conditions [105]. This functional duality is achieved via three orthogonal axes. First, melatonin buffers the redox environment [106], maintaining matrix viscosity within a window permissive for physiological phase separation [94]. Second, it exerts biophysical control over condensate scaffolds, employing multivalent π-π, π-cation, and electrostatic forces to interfere with condensate-interacting regions [105]. Third, melatonin synergizes with adenosine triphosphate (ATP) to modulate condensate solubility; its indole ring participates in van der Waals π-π stacking with the adenosine moiety of ATP, elevating their combined dipole moment and strengthening hydrogen bonding with the hydration shell [107]. By biasing the kinetic landscape of BCs, melatonin ensures optimal dynamics by re-fluidizing arrested condensates or inhibiting nascent assemblies to prevent the premature aging of BCs driven by pathological over-activation (Figure 7). Since melatonin modulates LLPS, resulting in changes in the redox environment, its antioxidant actions may be an essential agent to support and maintain this process in both the membranes and cytosol (see below).

## 7. Melatonin, Oxidative Load, Lipid Rafts and Mitochondrial Lipid Microdomains

Melatonin, oxidative stress, and lipid rafts form an interconnected physicochemical axis that defines how cells maintain membrane integrity, signaling fidelity, and resilience under redox pressure [93,108]. Melatonin partitions directly into lipid bilayers, where it interacts with cholesterol-rich lipid rafts; these transient microdomains organize receptors, ion channels, and signaling complexes essential for optimal cellular homeostasis [109]. Recent data show that melatonin modulates the structural dynamics of these rafts, stabilizing their organization across micro- and nanoscale distances and preserving membrane fluidity even under conditions of oxidative stress which threaten to disrupt raft architecture. ROS preferentially target PUFA within rafts, initiating lipid peroxidation that increases membrane viscosity, destabilizes raft-associated proteins, and impairs signaling pathways. Melatonin counters this through a dual mechanism: direct scavenging of ROS, thereby interrupting lipid peroxidation chain reactions [110,111,112]. Secondly, this protection is also supplied by metabolites, e.g., AMK, AFMK and others, which are generated during melatonin’s antioxidant cascade. Some of the metabolites are better than melatonin itself in derailing the lipid peroxidation chain reaction [113]. By reducing membrane viscosity and preventing oxidative fragmentation of raft lipids, melatonin preserves the lateral heterogeneity required for raft-dependent processes, including endocytosis, immune regulation, and mitochondria–plasma membrane communication [12,110]. In addition to direct radical scavenging, the protective effect of melatonin extends to its ability to modulate mitochondrial function by reducing ROS generation at the source, thereby also lowering the oxidative burden in membrane microdomains; this process has been designated radical avoidance and is achieved when melatonin increases the efficiency of electron flow through the respiratory chain [114].

As oxidative stress is a major driver of lipid raft disruption in aging, neurodegeneration, and cancer, melatonin’s capacity to maintain raft integrity positions it as a unique membrane-active antioxidant that safeguards both the physical structure and functional signaling roles of lipid rafts [115,116,117]. In this integrated framework, melatonin emerges not only as a defender against oxidative damage but also as a regulator of membrane organization, ensuring that lipid rafts remain functional hubs for cellular communication even under redox stress [12,110]. The actions of melatonin in preserving the architecture and function of lipid rafts are receptor independent.

Although mitochondrial membranes do not possess lipid rafts identical to those in the plasma membrane, mitochondria do contain raft-like microdomains. In the IMM, cardiolipin can form ordered microdomains analogous to those in the plasma membrane [118,119]. Cardiolipin is a critical phospholipid that plays an essential role in organizing respiratory chain complexes [120]. Oxidation of cardiolipin has a major negative impact on electron flow through the ETC and contributes in a major way to oxidative damage and to apoptosis. Melatonin reduces cardiolipin oxidation, preserves OXPHOS and inhibits programmed cell death in normal cells [13,121].

## 8. Mitochondrial Melatonin: Synthesis, Uptake, Function, and Redox Homeostasis

Even before a functional association was established between melatonin and mitochondria, cytochemical evidence identified these organelles as being the potential intracellular site of melatonin synthesis [122]. Although the quality of the published electron microscopic images is not optimal, the findings of this group clearly showed that acetyl serotonin transferase (currently known as N-acetyltransferase, which is rate limiting in melatonin production) is situated in the mitochondria, indicating these organelles are a major location of melatonin production, at least in pinealocytes. For cytochemical identification, the authors used a copper ferricyanide cytochemical method to identify the enzyme with the reaction product being almost exclusively confined to mitochondria; the pineal tissues were collected from rats during the night.

Roughly 25 years thereafter, publications definitively linking melatonin to mitochondrial function were published. The research group that initially contributed most significantly to defining the functional relationship of melatonin’s modulation to mitochondrial physiology is that of Acuna Castroviejo and colleagues [123]. Their seminal work suggested mitochondria as a primary melatonin target, showing that the indoleamine boosts complex I and IV activities, preserves glutathione, limits nitric-oxide-driven damage, and promotes ATP production. They also documented melatonin’s genomic effects within mitochondria, establishing its role in sustaining bioenergetic stability and preventing oxidative injury [124,125,126].

**Figure 8:**
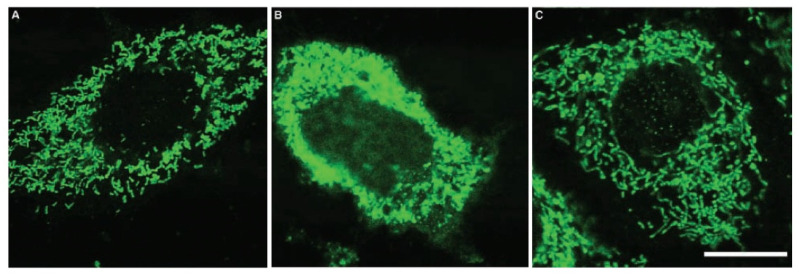
**Effect of melatonin on cytochrome c release from H_2_O_2_-treated astrocytes.** (**A**) On the left is an image of a control astrocyte which shows that cytochrome c (green fluorescence) is confined to the mitochondria. (**B**) The center is an astrocyte exposed to H_2_O_2_ for 10 min showing cytochrome c fluorescence throughout the cytosol and also in the nucleus. (**C**) The image on the right is of an astrocyte incubated in a solution of H_2_O_2_ but also having received supplemental melatonin. As in the control cell, cytochrome c is restricted to the mitochondria, proving that the permeability of the mitochondrial membranes was not perturbed. Figure provided by MJ Jou and printed with permission.

Concurrently, Jou [127] examined melatonin’s action at the mitochondria level with the aid of time-lapse conventional, confocal, and multiphoton fluorescent imaging microscopy coupled with a noninvasive mitochondria-targeted fluorescent probe. Using these techniques, they visualized that the indoleamine protects astrocyte mitochondria from Ca^2+^-triggered permeability transition, H_2_O_2_-induced reactive oxygen species formation, and apoptosis. The findings of these reports affirmed melatonin as a mitochondria-targeted antioxidant whose uptake and actions preserve organelle integrity and redox balance across several stressful conditions. The ability of melatonin to protect against ROS-mediated oxidative damage was obvious in the measurement of free-radical-dependent fluorescence in mitochondria and the release of cytochrome c, which is normally tightly associated with cardiolipin in the IMM, from mitochondria damaged by H_2_O_2_ (Figure 8) [128]. Melatonin proved more effective than vitamin E in protecting the mitochondria from ROS-induced damage. The release of cytochrome c into the cytosol requires permeabilization of the OMM, which is a consequence of BAX/BAK oligomerization resulting in the formation of pores through which cytochrome c escapes, eventually causing apoptosis. Each of these events are suppressed by melatonin.

Jou and co-workers [129] subsequently tested the efficacy of cyclic 3-hydroxymelatonin and AFMK, metabolites of melatonin formed during its scavenging cascade, and noted that they also reduced mitochondrial oxidative stress and stabilized mPTP. In more recent years, there have been numerous reports published by a wide variety of investigators that document the capacity of melatonin to modulate mitochondrial homeostasis, including its ability to influence Warburg-type metabolism via the disinhibition of mitochondrial pyruvate dehydrogenase and upregulation of SIRT3 [130].

In 2014, Acuna-Castroviejo and co-workers [131] published a comprehensive review in which the extrapineal tissues that likely synthesize melatonin were summarized. The large number of melatonin-containing vertebrate tissues that are listed in this survey is consistent with the presence of melatonin in non-vertebrate species, as well as in plants, all of which lack a pineal gland [132,133]. Since non-vertebrate species evolved eons before the vertebrates and the pineal gland, melatonin evolution predates the appearance of the pineal gland by an estimated two billion years [134]. In plants as well, melatonin is present in high concentrations even though these species are obviously devoid of any organs similar to those in vertebrates [135].

**Figure 9:**
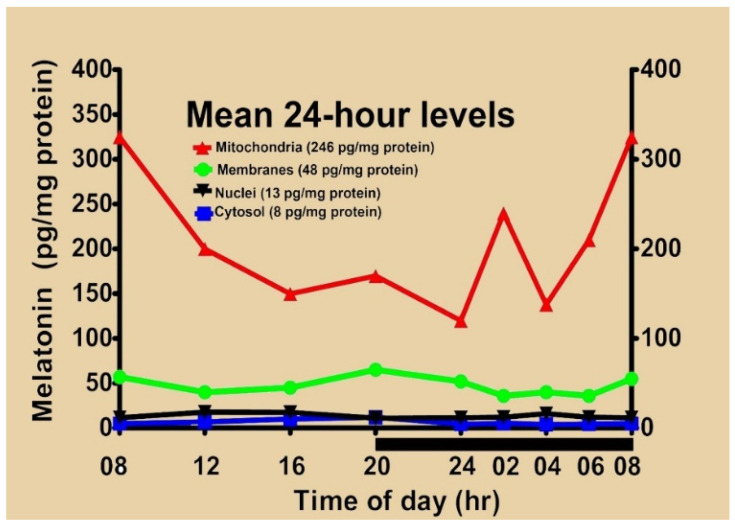
**Melatonin concentrations in subcellular fractions purified from rat cerebral cortices over a 24 h light/dark cycle**. The values among the cell fractions clearly varied widely, with the mitochondria having the highest concentration followed by membranes. The average 24 melatonin levels for each fraction are also listed. Unlike in the pineal gland, there was no circadian variation in the level of melatonin in any fraction, indicating the melatonin was not a result of uptake of circulating melatonin released from the pineal gland. Moreover, these measured values were not reduced in animals that had been pinealectomized three weeks earlier. The variations observed, such as those seen in the mitochondrial fractions, are thought to be a function of local production verses usage as a radical scavenger at the time of measurement. Similar data in terms of differential concentrations were noted in liver cells collected at the same time points. The black bar identifies the dark period. Figure restructured and published with permission [50].

The first biochemical documentation of melatonin in isolated mitochondria was provided by Venegas and colleagues [50]. In this report, brain cerebral cortices and liver cells were intermittently obtained from mice over a 24 h period. After the tissues were homogenized, the following subcellular compartments were isolated: membranes, mitochondria, nuclei and cytosol. As illustrated in Figure 9, under basal conditions, the highest levels of melatonin were measured in mitochondria followed by membranes, nuclei and the cytosol. Also, although there were some fluctuations in melatonin levels in each compartment over the light:dark period, no discernible rhythms were noted. The average concentration of melatonin in mitochondria (246 pg/mg protein) was roughly 5-fold higher than that in membranes, which was roughly 48 pg/mg protein at each time point. Much lower levels were found in the cytosol and nuclei. Neither mitochondrial, nuclear nor cytosolic levels were altered in animals that had been pinealectomized earlier, indicating the cellular melatonin was clearly not of pineal origin. Paradoxically, rather than being diminished, membrane levels of melatonin exclusively increased in the pinealectomized mice, suggesting compensatory elevation of intrinsic melatonin production.

Based on the accumulating data, more than a decade ago, we hypothesized that melatonin synthesis normally occurs in the mitochondria of both animal and plant cells and also in the chloroplasts of plant tissues [136,137]. This prediction was made on the basis of the studies highlighting melatonin’s actions in mitochondria, the existence of melatonin in tissues of species that lack a pineal gland, its presence in the mitochondria of mammalian cells, and, importantly, the development of these organelles from melatonin-synthesizing microbes that were engulfed by early eukaryotes and which over eons eventually transitioned into mitochondria and chloroplasts [134,135,137]. This also means that melatonin predated the emergence of the pineal gland, which is exclusive to vertebrates, by about two billion years [51].

Further evidence supporting the synthesis in mitochondria and chloroplasts was forthcoming soon thereafter. In the most complete study to date, Suofu and colleagues [56] identified proteins for the two enzymes that convert serotonin to melatonin, i.e., AANAT and N-acetyl serotonin methyltransferase (ASMT), in neural cell mitochondria and also proved that these organelles converted deuterated serotonin to deuterated melatonin. A year later, Quintela and coworkers [138] identified melatonin synthesis in the mitochondria of rat choroid plexus cells. Suofu and colleagues [56] observed that melatonin levels do not exhibit a circadian rhythm in brain mitochondria, affirming the observations of Venegas and co-workers [50] and distinguishing it from the cycle in the pineal gland. Melatonin production in chloroplasts was first uncovered by Zheng and colleagues and later confirmed in many plant species [135,139].

The first experimental findings that provide support for the hypothesis that melatonin may be synthesized in the mitochondria of all mammalian cells, not only brain cells and hepatocytes, was provided by He and coworkers [140]. Based on the observations that had already reported the presence of melatonin in intact oocytes [141], and knowing that melatonin plays pivotal roles in safeguarding the oocyte from oxidative damage and promoting its maturation, He and coworkers [140] examined whether purified mitochondria obtained from the oocytes of hormone-induced superovulated mice had the capacity to synthesize melatonin. When incubated with serotonin, a necessary precursor of melatonin, the level of melatonin increased markedly in the incubation medium. The findings that melatonin production occurs in isolated oocyte mitochondria support the likelihood that the mitochondria of all vertebrate’s somatic and germline cells have retained the synthetic capacity to generate melatonin given that these organelles are all descendants of those in the oocyte. Thus, it is reasonable to assume that since oocyte mitochondria produce melatonin, this critical action would have been preserved and inherited by all cells in the developed organism [130].

Similar but more extensive observations were subsequently reported. Using porcine oocytes, Zhu and colleagues [142] affirmed that mitochondria are the essential sites of endogenous melatonin production and that the indoleamine has critical roles in curtailing ROS-mediated damage, enhancing the activity of the respiratory ETC, and supporting ATP production and mitochondrial biogenesis. When mitochondrial melatonin production was inhibited with the use of siRNA to knockdown *AANAT*, the oocytes suffered from delayed in vitro maturation and reduced ATP production. These changes were restored when the cells were incubated with supplemental melatonin. As with the earlier study using mouse oocyte mitochondria, these findings are again in accordance with melatonin’s synthetic activity being passed to all cells in the developed organism. The ability of melatonin, presumably locally produced in mitochondria, to improve oocyte quality has also been observed in human oocytes [143].

While many tissues contain melatonin [131], the number of cells in which melatonin synthesis has been directly linked to mitochondria is obviously small. It is unlikely, however, that the substantial levels of melatonin in the tissues where it has been measured is not a result of its uptake from the blood after its release from the pineal rather than being intrinsically produced. The pineal gland in all species is miniscule and, moreover, it exclusively synthesizes melatonin during the dark period, with circulating values usually falling in the pg/mL range, concentrations that would be insufficient to seed all other cells with this indoleamine. Also, all species that lack a pineal or a homolog of this organ contain melatonin, e.g., invertebrates and plants.

**Figure 10:**
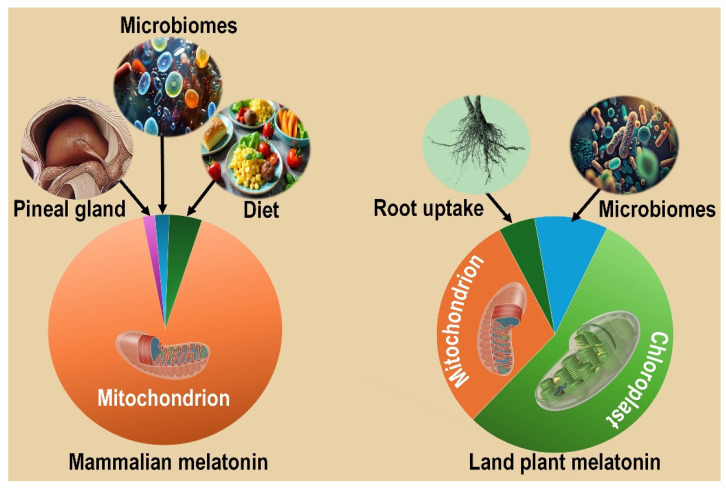
**Derivation of melatonin in vertebrates and plants with estimates of the contribution of each source.** Of the total quantity of melatonin produced in mammals, the pineal gland contributes very little because of its small size and is unique production exclusively during the night. While the amount of melatonin from the pineal gland is small, it is critically important since it is released into blood and cerebrospinal fluid (CSF) and modulates circadian rhythms. In mammals, the largest portion of melatonin is from mitochondrial synthesis in every cell. This melatonin forms the non-releasable pool since it does not enter the blood, presumably since it would interfere with the circadian action of pineal-released melatonin; rather, it is used locally as an antioxidant and cell protector. Small amounts of melatonin are also ingested in the diet, and some are produced in microbes of the gut and other microbiota. In plants, both mitochondria and chloroplasts synthesize non-releasable melatonin. Additionally, melatonin is synthesized by plant microbiomes, e.g., rhizosphere, etc., and it is also absorbed through the roots.

In addition to its intrinsic synthesis in mitochondria, there are other sources in addition to the pineal gland that could contribute to the wide tissue distribution of melatonin in vertebrates. Most products that are consumed by vertebrates contain melatonin which may influence circulating melatonin levels. The bioavailability of orally ingested melatonin, however, is low (3–30%) and highly variable [144] and the reported induced changes in circulating melatonin levels is small and transient [145]. The gut microbiota is also a potential source of melatonin in somatic cells given that some of these microbes produce the indoleamine, which could be released and gain access to the systemic circulation [59,146], as recently suggested [147]. Based on the most compelling published data, however, the capacity of individual mitochondria to synthesize melatonin is the most likely major source of this agent in the animal and plant mitochondrial matrix and in plant chloroplasts (Figure 10). Finally, perhaps the strongest evidence for this conclusion is based on studies showing that bacteria, the forerunners of mitochondria in eukaryote cells, possess the required enzymes to generate melatonin [148,149].

Taking supplements of melatonin, which provide much larger amounts than the quantity derived from the diet, do enter cells, as shown in the data discussed earlier in this section. Melatonin is small, uncharged and has both lipophilic (indole ring) and hydrophilic (acetamide) components such that it partitions directly into lipid bilayers, allowing it to diffuse across both cell and mitochondrial membranes [109,110,150]. Additionally, via its conventional receptors, MT1 and MT2, melatonin undergoes ligand-mediated endocytosis, assisting its entrance into cells [151]. Also, by means of transporter-assisted uptake through the GLUT 4 receptor and/or oligopeptide PEPT1/2 transporters, melatonin gains access to cells and mitochondria [152,153]. While the passive diffusion route may be the dominant path for the entrance of melatonin into cells and mitochondria, this is not unequivocally established.

## 9. Mitochondrial Melatonin: Functional Inducibility

There is substantial literature related to the upregulation of melatonin in response to both abiotic and biotic stressors across a wide range of plant species [132,135,154]. As in animals, melatonin in plants functions as a multifaceted direct free radical scavenger and indirect antioxidant [155]. Since these stressful challenges invariably induce high ROS generation, the simultaneously elevated production of the antioxidant melatonin provides essential protection from molecular damage which would otherwise be substantial. This is especially important in plants that are under stress due to excessive temperature extremes, drought or water logging since they are sessile and cannot avoid these insults. This contrasts with animals that can behaviorally adjust to evade some stressors. As a result, the action of stress-induced augmented melatonin production in extrapineal cells of animals is less clear than in plants. Moreover, upregulation of pineal melatonin synthesis and release under these conditions would not serve a useful purpose in terms of protecting against oxidative stress in peripheral tissues given that melatonin must be onsite when and where free radicals originate (Figure 1).

Recently, non-visible near-infrared radiation (NIR) (600–1000 nm wavelength) has attracted attention as a potential stimulus for melatonin production in extrapineal cells because it penetrates several centimeters into tissues and it induces ROS generation [156]. NIR photons are absorbed by Complex IV (cytochrome c oxidase) of the ETC, thereby promoting ATP production while also advancing ROS production. Via a nitric-oxide-mediated pathway, mitochondrial AANAT is phosphorylated by the upregulated protein kinase A, which is also present in the mitochondria. We proposed that this sequence of events would likely stabilize pAANAT due to the recruitment of the chaperone 14-3-3 potentially enhancing mitochondrial melatonin synthesis [157]. The idea that NIR impacts mitochondrial melatonin synthesis is especially relevant to the current trend to delete these wavelengths from artificial light sources; this deletion could remove a major stimulus for the generation of a potent antioxidant and cell protector, which may contribute to accelerated aging of the effected tissues. Also, since NIR has become increasingly used in a treatment known as photobiomodulation [158,159,160], there is a pressing need to more reliably define the role of NIR in modulating extrapineal melatonin production, e.g., in skin cells which are already known to produce this important indoleamine [161]. Some of the reported benefits of photobiomodulation may be a consequence of the activation of mitochondrial melatonin synthesis in the exposed cells.

The inducibility of melatonin in cells that combat airborne toxins and/or ingested microorganisms has been examined [162]. The cells of interest have been resident macrophages and microglia, elements that serve as the first line of defense against pathogen-associated molecular patterns (PAMPs) and damage-associated molecular patterns (DAMPs), which activate pattern recognition receptors (PRRs), triggering inflammatory signaling cascades. Consistent with melatonin’s acknowledged anti-inflammatory actions [163,164], melatonin synthesis is upregulated in macrophages where it has been measured, which aids in the suppression of the innate inflammatory response. These studies have been directed primarily to alveolar macrophages. Associated with the activation of melatonin production at these extrapineal sites, Pontes and colleagues [165] note that humans with elevated circulating levels of inflammatory tumor necrosis factor (TNF) exhibit reduced nocturnal circulating melatonin concentrations. Thus, the induced rise in peripheral macrophage melatonin levels seems to be accompanied by suppressed pineal circadian melatonin synthesis and release. The chain of events by which macrophage-produced melatonin specifically contributes to defensive processes against biotic and abiotic stress involves inhibition of the NF-κB and related processes [162].

Strenuous physical exercise induces a minor and short-term rise in circulating melatonin, but its site or origin has not been determined [156,166,167]. While it was always assumed that the increases in blood melatonin levels in these individuals were derived from the pineal gland, in view of more recent information, it seems more likely that the melatonin rises were a result of the leakage of melatonin that was produced peripherally. Peripheral cells do not normally release melatonin into the circulation since it could disrupt normal circadian rhythmicity [51]. The weak increases in circulating melatonin values may indicate that, within cellular mitochondria of the peripheral cells, e.g., in skeletal muscle cells, the increases were much larger. A curious observation reported by Trojani and coworkers [168] implies that stress may hasten the uptake of circulating melatonin into cells, possibly for the purpose of negating the damaging actions of ROS which are produced in excess during extreme exercise.

The ROS-driven mechanisms leading to the functional upregulation of mitochondrial melatonin production may involve several different processes. Thus, Nrf2, which is activated under high oxidative stress conditions, elevates the transcription of AANAT and ASMT. Indirect phosphorylation and stabilization of AANAT occurs during short-term rises in mitochondrial Ca^2+^ levels, perturbations in the mitochondrial membrane potential alter substrate flux, mitochondrial acetyl CoA availability may increase in response to ROS, which aids in promoting AANAT activity, and SIRT3-dependent deacetylation stabilizes melatonin-generating enzymes [169].

## 10. Conclusions: Final Thoughts Distilled

This review summarizes information related to the diverse methodologies that were used to repeatedly confirm the radical scavenging actions of melatonin. Using each of these platforms, the data obtained document that melatonin in pure chemical systems, as well as in cultured and in vivo cells, uniformly scavenges damaging free radicals and significantly reduces oxidative stress to critical molecules including DNA, lipids and proteins. Importantly, this action is also seen at the level of mitochondria, an organelle that is frequently severely damaged by ROS since their production is a consequence of mitochondrial metabolism. This cataloging was done in part since there are still some researchers who question whether melatonin has direct ROS scavenging activities [7,8,9]. Since the data were obtained in multiple research laboratories using numerous techniques, the findings are consistent with melatonin being a receptor-independent direct ROS scavenger. The scavenging actions do not rely on the interaction of melatonin with a receptor since this reaction occurs in pure chemical systems and in synthetic model lipid membranes which are devoid of any receptors (see Section 7 above). However, there are many other actions of melatonin that are unequivocally related to its involvement with both membrane and possibly intracellular receptors. For example, the upregulation of antioxidative enzymes and the promotion of glutathione production are believed to follow melatonin’s interaction with membrane and/or nuclear receptors.

Of critical importance is the presence of melatonin in the mitochondrial matrix given that the ETC is a major site of free radical generation. Moreover, mitochondria, in addition to being the source of OXPHOS and ATP synthesis, houses the citric acid cycle which provides NADH and FADH_2_ in support of the ETC complexes, influences metabolic pathways by modulating β-oxidation of fatty acids and impacting amino acid catabolism, regulates apoptosis by managing BAX/BAK pores in the OMM and sequestering cytochrome c and other apoptogenic factors in the IMM, maintains calcium flux via the calcium uniporter and VDAC channel, and modulates innate immune signaling, among other important functions [170,171,172,173,174]. Each of these functions is influenced by the state of the redox balance in these organelles [175,176,177]. Thus, the detoxification of the abundant locally produced ROS to maintain the optimal welfare of mitochondria and the survival of the cells themselves is of utmost importance. Due to its synthesis in the mitochondria of possibly all cells, melatonin is ideally positioned to prevent the onsite molecular damage inflicted by rapidly damaging and short-lived radical products [134]. While the current review primarily describes the mechanisms by which melatonin and several of its metabolites directly neutralize ROS, melatonin also provides a multi-layered antioxidative system which includes a variety of distinct actions that enhance the efficacy by which it attenuates oxidative stress and preserves cellular and organismal health [178,179,180,181]. These functions include upregulation of antioxidative enzymes, including mitochondrial superoxide dismutase [182], catalase [183], and glutathione peroxidase [184]; downregulation of pro-oxidative enzymes [185]; stimulation of γ-glutamylcysteine synthetase, which rate-limits glutathione production, an important antioxidant in its own right [186,187]; synergism with conventionally ingested antioxidants such as vitamin E [188]; reduction in electron leakage (radical avoidance) from the ETC [114]; and others.

Given the numerous fundamental functions of mitochondria which ensure the health and survival of cells, it is obviously imperative that they be maintained in their prime physiological state. A major self-imposed threat to their ideal operation is their excessive generation of ROS via the ETC and other processes. Due to the indiscriminate and persistent damage by ROS to critical components of these organelles, and, by association, the cells in which they reside, they are continually in jeopardy of failure, which contributes to aging and diseases. The synthesis of a versatile and potent antioxidant, melatonin, in mitochondria could play a substantial role in safeguarding these organelles from molecular damage and dyshomeostasis. The findings summarized in this review, if validated for the mitochondria of all cells, could be a major means to protect these organelles and reduce the frequency or severity of mitochondria-related diseases, of which there are many [189,190].

Melatonin synthesis has been identified in the mitochondria of every cell in which it has been examined and in several microbial species, there are evolutionary precursors of mitochondria and chloroplasts in both eukaryotic plants and animals [134,191,192]. Due to its diverse molecular skills to incapacitate ROS, the presence of melatonin in mitochondria would be metabolically advantageous. Likewise, mitochondrial physiology would benefit if melatonin concentrations were adjusted in accordance with the quantity of ROS being generated. In plants, it has been repeatedly confirmed that melatonin production is quickly upregulated in response to ROS-generating abiotic and biotic stressors, thereby preserving plant survival [154]. In eukaryotic animals, the evidence for the induction of melatonin in peripheral organs in response to stress is less compelling, although blood concentrations rise transiently [156,166,167]. Assessing potential changes in peripheral cell mitochondria following challenges that elevate ROS formation could provide essential information regarding melatonin as protector of mitochondrial welfare. These measurements may be assisted by using a newly designed colorimetric assay with a detection limit of melatonin as low as 0.6 nM [193].

Mitochondria are essential for meeting the metabolic demands of all cells. Contact-dependent and non-contact-dependent pathways for intercellular mitochondrial transport are fundamental processes that cells have evolved to aid in functional mitochondrial maintenance by enhancing bioenergetics, shaping redox homeostasis, and improving cellular resilience. Mitochondria are transported between cells via tunneling nanotubes (TNTs), extracellular vesicles, and gap junction channels, and they also strengthen cells physiologically because of cell fusion [194,195,196]. These processes are highly regulated, and the mitochondria carry with them the enzymatic machinery for melatonin synthesis, a molecule that is highly beneficial in preserving the function of normal cells and restoring the physiology of faltering cells [197]. Moreover, melatonin promotes the rate of transfer and the number of mitochondria moves between cells by TNTs [198].

Within the last decade, physical mitochondrial transplantation has emerged as a technology for restoring normally functioning mitochondria to damaged tissues [199,200,201]. This process involves isolating viable mitochondria from normal donor cells and injecting them directly into damaged or diseased tissue (or into the blood stream) from where they may be actively internalized by dysfunctional cells. The injected healthy mitochondria assist in re-establishing energy production and, given that the internalized mitochondria may produce melatonin, they could also likely buffer the high oxidative environment of the recipient cells, which is common in diseased and in injured cells, thereby improving their survival. Some injected mitochondria do not tolerate isolation or injection procedures. The current authors surmise that exposing the isolated mitochondria to supplemental melatonin would improve their survival. For example, previous reports established that transplanted organs exhibit increase viability when they are incubated in a melatonin-containing solution before transplantation, presumably because they are better equipped to overcome oxidative damage [202].

The use of melatonin by humans as either a prescription drug or as an over-the-counter supplement is common, with daily doses varying widely (from one to hundreds of mg daily). The molecule has a very high safely profile, with only a few difficult-to-quantify subjective side effects occasionally being mentioned, e.g., dizziness, sleepiness, and headache. Melatonin has been given at a dose of 1000 mg daily for 30 days with no evidence of toxicity [203] and many other authors have noted its minimal or essential lack of side effects when ingested daily by humans [204,205,206]. When used to defer or treat diseases, the human-equivalent dose allometrically determined on the basis of results from animal studies is estimated to be 1.0–1.5 mg per kg of body weight [207].

Oral bioavailability varies widely depending on the hepatic activity of the melatonin-metabolizing enzyme CYP1A2 and other factors with commonly reported bioavailability ranges of 10–30% [206]; this also varies with the release form used [208]. By avoiding the first-pass effect, bioavailability is much higher when melatonin is given via other routes, e.g., sublingually or intravenously [206]. Also, combining melatonin with different nanoparticles greatly increases both its bioavailability and efficacy [209,210,211].

**Figure 11:**
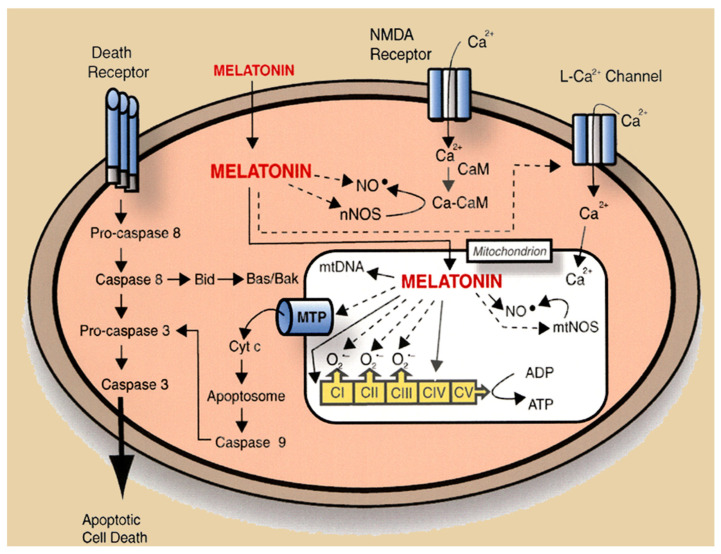
**Actions of melatonin following its uptake into cells and into mitochondria.** In the mitochondria, melatonin functions as an efficient scavenger of free radicals (represented here by O_2_^∙−^), which are prominently a consequence of electrons escaping the electron transport chain (CI-CIV). Melatonin also inhibits the release of cytochrome c (cyt c) which impacts both intrinsically and extrinsically regulated apoptosis via the pathways indicated. Melatonin is a physiologically targeted antioxidant. CaM = calmodulin; mNOS = mitochondrial nitric oxide synthetase; MTP = mitochondrial transition pore; nNOS = neuronal nitric oxide synthetase.

For exogenously administered melatonin to curtail mitochondrial ROS-mediated damage, it must enter these organelles. While there are no direct measures of melatonin concentrations in mitochondria after its in vivo administration, the physiological evidence that melatonin readily enters the mitochondria is persuasive. As reviewed in the earlier sections of this report, melatonin quickly alters ETC activity and ATP synthesis and negates oxygen-radical-mediated molecular damage and cytochrome c release, among many other changes, including altering mitophagy, biogenesis, and apoptosis (Figure 11). Loading antioxidants into mitochondria has been a major interest of pharmaceutical scientists for at least two decades. One established method for achieving this is with the use of the TTP^+^ [212,213]. Since it possesses a positively charged phosphonium core functional group and lipophilic phenyl rings, it has proven especially effective in crossing mitochondrial membranes. This agent has become a universal delivery vector for increasing antioxidant levels in the mitochondria; examples include its combination with vitamin E (MitoE) and ubiquinone (CoQ10) (MitoQ) [136,214,215]. While melatonin is already considered a mitochondria-targeted antioxidant [216], it can be further optimized to do so by linking it to the TTP^+^, with its efficacy at the mitochondrial level being immensely exaggerated up to 1000-fold [217]. The TTP–melatonin (Mito-MEL) conjugate could be highly significant for melatonin’s usefulness in modulating energetic activities, redox homeostasis of mitochondria, and countering disease processes in future clinical trials.

## Data Availability

There are no new raw data associated with this article. All data reported in this paper will be shared by the lead contact upon request.

## Abbreviations

The following abbreviations are used in this manuscript:

·OH	Hydroxyl radical
3-OHM	3-Hydroxymelatonin
4OHM	4-Hydroxymelatonin
5-HT	5-Hydroxytryptamine
5-MT	5-Methoxytryptamine
6-cMEL	6-Chloromelatonin
6OHM	6-Hydroxymelatonin
AANAT	Arylalkylamine N- acetyltransferase
AANAT-KO	Arylalkylamine N-acetyltransferase knock out
ABTS	2,2′-azino-bis(3-ethylbenzothiazoline-6-sulfonic acid)
ABTS·^+^	ABTS radical cation (the one-electron oxidized form)
AFMK	N1-acetyl-N2-formyl-5-methoxykynuramine
AMK	N1-acetyl-5-methoxykynuramine
AOX-E	Antioxidant enzyme modulators
AOX-I	Primary antioxidant activity or interception
AOX-II	Secondary antioxidant activity
AOX-III	Tertiary antioxidant activity
Asc^−^	Ascorbate ion
ASMT	Acetyl serotonin O-methyltransferase
ATP	Adenosine triphosphate
BAK	Bcl-2 homologous antagonist/killer
BAX	Bcl-2-associated X protein
BCs	Biomolecular condensates
CCl_3_O_2_^·^	Trichloromethylperoxyl radical
CSF	Cerebrospinal fluid
DEPMPO	5-(diethoxyphosphoryl)-5-methyl-1-pyrroline-N-oxide
DFT	Density functional theory
DMPO	5,5-dimethyl-1-pyrroline-N-oxide
ESR	Electron spin resonance spectroscopy
ETC	Electron transport chain
fHAT	Formal hydrogen atom transfer
FMN	Flavin mononucleotide
GLUT 4	Glucose transporter type 4
H_2_O_2_	Hydrogen peroxide
HOCl	Hypochlorous acid
IC50	Half-maximal inhibitory concentration
IMM	Inner mitochondrial membrane
IMS	Intermembrane space
KEAP1	Kelch-like ECH-associated protein 1
KN	Kynuramine
LDH	Lactate dehydrogenase
mPTP	Mitochondrial permeability transition pore
N2a	Neuro-2a mouse neuroblastoma cell line
NAD+	Nicotinamide adenine dinucleotide (oxidized form)
NADH	Nicotinamide adenine dinucleotide (reduced form)
NF-κB	Nuclear factor-κB
NIR	Near-infrared radiation
nNOS	Neuronal nitric oxide synthase
NO^∙^	Nitric oxide
NO_2_^−^	Nitrite anion
Nrf2	Nuclear factor erythroid 2-related factor 2
O_2_	Oxygen
O_2_^∙−^	Superoxide anion radical
OGD	Oxygen glucose deprivation
ONOO^−^	Peroxynitrite anion
ONOOH	Peroxynitrous acid
OPTN	Optineurin
OXPHOS	Oxidative phosphorylation
PAMPs	Pathogen-associated molecular patterns
PEPT1/2	Peptide transporter 1 or 2
PPARα	Peroxisome proliferator-activated receptor alpha
PRRs	Pattern recognition receptors
PUFA	Polyunsaturated fatty acid
Q (Coenzyme Q)	Ubiquinone
QH_2_	Ubiquinol
QM-ORSA	Quantum mechanics-based test for overall free radical scavenging activity
RNS	Reactive nitrogen species
ROO·	Peroxyl radical
ROS	Reactive oxygen species
SHATD	Sequential hydrogen atom transfer dihydration
SIRT3	Sirtuin-3, a mitochondrial deacetylase
SSI	Similarity interaction score
TF	Transcription factor
TNF	Tumor necrosis factor
TNT	Tunneling nanotubes
TrxR	Thioredoxin reductase
TST	Transition state theory
TTP+	Triphenylphosphonium cation
UCP2	Uncoupling protein 2
UV	Ultraviolet light
WT	Wild type
